# Source-Identifying Biomarker Ions between Environmental and Clinical *Burkholderia pseudomallei* Using Whole-Cell Matrix-Assisted Laser Desorption/Ionization Time-of-Flight Mass Spectrometry (MALDI-TOF MS)

**DOI:** 10.1371/journal.pone.0099160

**Published:** 2014-06-10

**Authors:** Suthamat Niyompanich, Janthima Jaresitthikunchai, Kitima Srisanga, Sittiruk Roytrakul, Sumalee Tungpradabkul

**Affiliations:** 1 Department of Biochemistry, Faculty of Science, Mahidol University, Bangkok, Thailand; 2 National Center for Genetic Engineering and Biotechnology, Pathumthani, Thailand; George Mason University, United States of America

## Abstract

*Burkholderia pseudomallei* is the causative agent of melioidosis, which is an endemic disease in Northeast Thailand and Northern Australia. Environmental reservoirs, including wet soils and muddy water, serve as the major sources for contributing bacterial infection to both humans and animals. The whole-cell matrix-assisted laser desorption/ionization time-of-flight mass spectrometry (whole-cell MALDI-TOF MS) has recently been applied as a rapid, accurate, and high-throughput tool for clinical diagnosis and microbiological research. In this present study, we employed a whole-cell MALDI-TOF MS approach for assessing its potency in clustering a total of 11 different *B. pseudomallei* isolates (consisting of 5 environmental and 6 clinical isolates) with respect to their origins and to further investigate the source-identifying biomarker ions belonging to each bacterial group. The cluster analysis demonstrated that six out of eleven isolates were grouped correctly to their sources. Our results revealed a total of ten source-identifying biomarker ions, which exhibited statistically significant differences in peak intensity between average environmental and clinical mass spectra using ClinProTools software. Six out of ten mass ions were assigned as environmental-identifying biomarker ions (EIBIs), including, m/z 4,056, 4,214, 5,814, 7,545, 7,895, and 8,112, whereas the remaining four mass ions were defined as clinical-identifying biomarker ions (CIBIs) consisting of m/z 3,658, 6,322, 7,035, and 7,984. Hence, our findings represented, for the first time, the source-specific biomarkers of environmental and clinical *B. pseudomallei*.

## Introduction

Melioidosis is a serious, often fatal, human disease which is caused by a motile, Gram-negative bacillus namely, *Burkholderia pseudomallei*. This disease is widely prevalent in tropical zones between latitudes 20°N and 20°S, which are commonly reported in Southeast Asia and Northern Australia [1]. These endemic areas have been illustrated with high mortality rates of about 40% [2]. *B. pseudomallei* is an environmental saprophyte which is normally found in wet soils and muddy waters contributing bacterial infection into humans and animals [3]. People who are directly in contact with soil and water contaminated with *B. pseudomallei* are affected by this disease [1], [4]. Cases of disease transmission among humans are rarely reported [3]. Clinical signs, representing in patients with melioidosis, vary from asymptomatic, localized acute or chronic pneumonia, and septicemia forms, which require antibiotic treatment for long periods [1], [5]. Currently, there are no vaccines available against this disease [2].

The epidemiological data of melioidosis in Thailand has revealed significantly higher infection rates in patients from northeastern regions than those in other parts of Thailand, in agreement with several reports from other countries including in Laos and Taiwan, regarding the relationship between occurrence of the disease and environmental exposure to *B. pseudomallei* [6]–[9]. Hence, melioidosis is considered as an environmental disease [10], owing to soil and water being observed to be important reservoirs for this organism. Isolates of *B. pseudomallei* from environmental and clinical sources are markedly diverse but some isolates from either group can be categorized into the identical molecular type [11]. Evidence from the studies by Haase et al have demonstrated that soil isolates mostly show less cytolethality compared to isolates from patients [12]. In contrast, Liew et al have investigated the enzyme profiling of environmental and clinical *B. pseudomallei* using the APIZYM system. The results have shown that bacteria from two sources secrete similar enzymes and the environmental isolates display higher protease activity than clinical isolates [13]. However, the association of virulence levels, according to their respective sources, is controversial and has yet been elucidated. Therefore, the source-identification of *B. pseudomallei* is essential not only for an implementing epidemiological strategy but also for surveillance, prevention and control of melioidosis.

Many studies have attempted to differentiate *B. pseudomallei* isolates from distinct origins by using various molecular typing tools including, ribotyping, pulsed-field gel electrophoresis (PFGE), restriction fragment length polymorphism (RFLP), randomly amplified polymorphic DNA (RAPD), multilocus enzyme electrophoresis (MEE), and multilocus sequence typing (MLST) [14]–[20]. In addition, a recent study by Bartpho et al has investigated the genomic islands (GIs) for use as the potential marker for distinguishing environmental and clinical isolates of *B. pseudomallei* on the basis of the microarray-based comparative genome hybridization (CGH) method [21]. But the limitations of these molecular approaches are that they are time-consuming, labour- and cost-intensive and require several steps to accomplish in the identification and typing of microorganisms. Thus, new analytical tools are needed to provide a better analysis [22].

In recent years, matrix-assisted laser desorption/ionization time-of-flight mass spectrometry (MALDI-TOF MS) is a well-established instrument used in routine clinical diagnosis and in various fields of microbiological research, including microbial systematics, environmental microbiology, and epidemiology [23], [24]. This is because it provides an ideal identification system and has more advantages over conventional methods, such as offering rapid, accurate, economical, and high throughput analysis [25], [26]. In principle, MALDI-TOF MS generates a unique mass spectrum as a fingerprint from whole-cell bacteria or crude extract. Obtained spectra can further be compared to the reference spectra in a database, resulting in the scores for identification at both the genus and species levels [22], [26], [27]. This technique also allows the identification at subspecies or strain level [28], [29] and can produce biomarkers calculated from particular algorithms which are specific for those species of interest [25]. MALDI-TOF MS has been used to identify and discriminate a wide range of microorganisms, including *Escherichia* [30], *Staphylococcus* [31], *Salmonella* [32], *Enterococcus* [33], *Candida* [34], *Lactococcus* [35], *Aeromonas* [36], *Vibrio* [37], and *Erwinia* [38]. Moreover, it is being introduced to utilize in biodefense applications by identifying the presence of biomarker mass ions in biological weapons (BW), comprising microorganisms and biotoxins [39]. The Centers for Disease Control (CDC) has designated *B. pseudomallei* as a category B agent due to its potential as a bioterrorism weapon [40]. The rapid and robust identification of *B. pseudomallei* based on MALDI-TOF is therefore required for early-warning and medical prevention of melioidosis. The recent study has applied whole-cell MALDI-TOF MS for identification and differentiation of *B. mallei* and *B. pseudomallei* [41]. However, there are no reports of anyone using whole-cell MALDI-TOF MS to discriminate between environmental and clinical isolates of *B. pseudomallei*. In this study, we have employed the whole-cell MALDI-TOF MS approach to examine its ability to group *B. pseudomallei* isolates according to their respective sources and investigate the source-specific biomarkers for distinguishing these isolates of different origins.

## Results

### Identification of *Burkholderia pseudomallei* isolates

**Figure 1:**
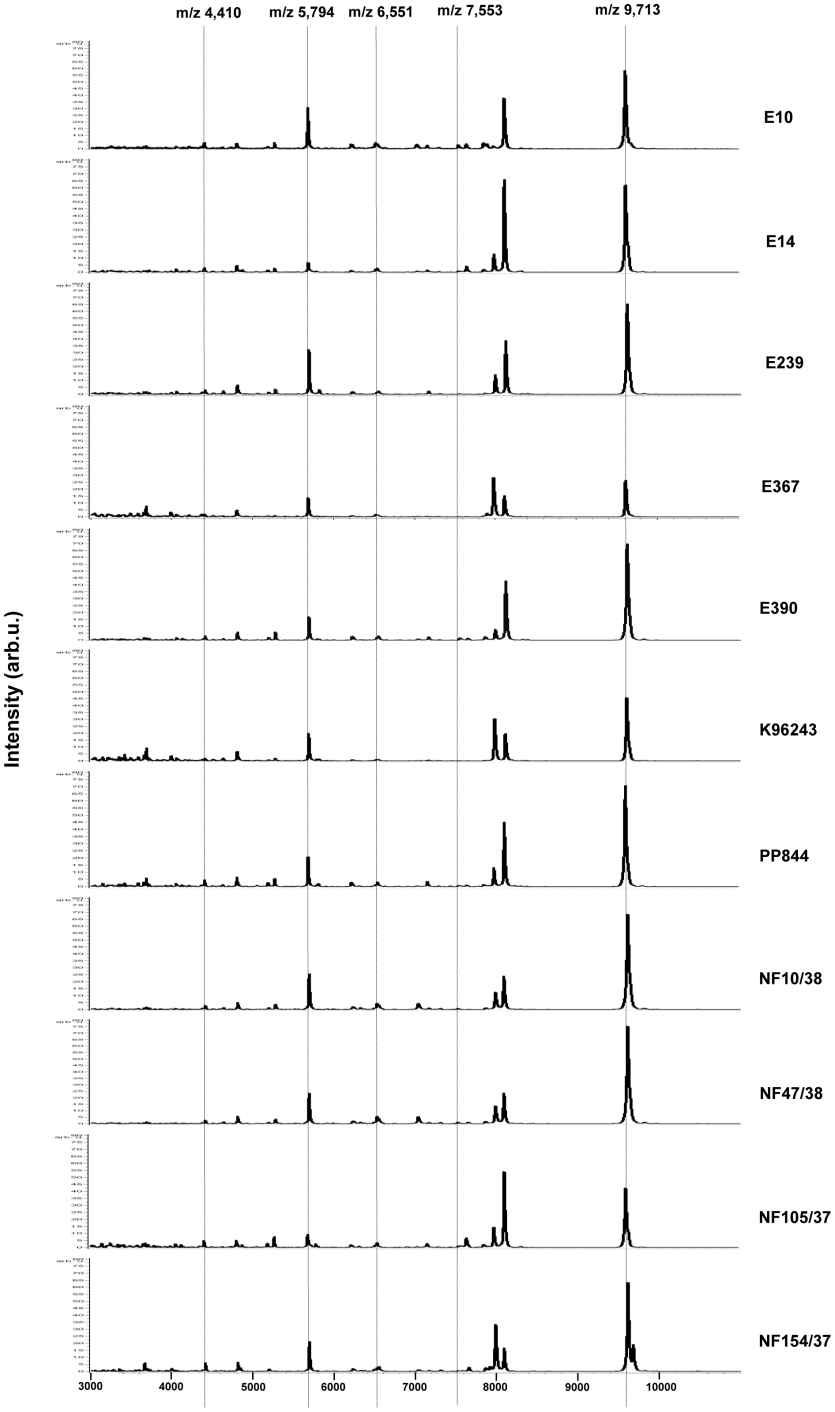
Average MALDI-TOF mass spectra of each *B. pseudomallei* isolates. Environmental (E10-E390) and clinical *B. pseudomallei* (K96243-NF154/37) samples displayed very similar peak patterns. All prominent biomarkers proposed by Karger et al for the identification of *B. pseudomallei* at m/z 4,410, 5,794, 6,551, 7,553, and 9,713 were detected in all mass spectra of tested strains in this study (vertical dashed lines).

To determine whether all environmental and clinical isolates were *B. pseudomallei* based on whole-cell MALDI-TOF MS method, we performed pattern matching and considered obtaining scores for identification using BioTyper 2.0 software. Generally, identification scores based on pattern matching imply reliable identification at the genus or species levels for any tested microorganism. Scores are obtained by comparison of tested mass fingerprints with the main spectral projections (MSPs) resulting in logarithmic scores between 0 (unrelated) to 3 (identical) [41]. Criteria for microorganism identification that are suggested by manufacturer are: (1) unreliable identification has a score < 1.7, (2) genus identification has a score between 1.7–1.9, and (3) species identification has a score ≥ 1.9 [42]. Since MSPs of *B. pseudomallei* were not available in our MALDI BioTyper library, we generated *B. pseudomallei* K96243 mass spectra as an in-house reference spectrum, by using BioTyper 2.0 to confirm identification of other tested isolates. All of the mass spectra queries collected from FlexAnalysis were matched against K96243 reference spectrum giving a score for each isolate as summarized in Table 1. The identification scores obtained were between 1.90–2.48, thus all samples in this study were confirmed, at species level, as *B. pseudomallei*. The average spectrum of each *B. pseudomallei* strain was generated by a combination of raw twenty mass spectra using the ClinProTools software (Figure 1). It could be observed that the mass patterns were similar among both environmental and clinical isolates. The recent study from Karger et al has shown the unique biomarkers for identifying *B. pseudomallei* and *B. mallei* using the intact cell MALDI-TOF method [41]. As the results from Karger et al show, the specific mass ions at 4,410, 5,794, 6,551, 7,553, and 9,713 are used as taxon-specific biomarkers in all *B. mallei* and *B. pseudomallei* samples. Mass peak at m/z 4,410 is commonly found in all nine *Burkholderia* species (*B. mallei*, *B. pseudomallei*, *B. thailandensis*, *B. ambifaria*, *B. cenocepacia*, *B. dolosa*, *B. glathe*, *B. multivorans*, and *B. stabilis*). Another important biomarker is m/z 9,713 which is used for differentiating the *Pseudomonas* group, including *B. mallei*, *B. pseudomallei*, and *B. thailandensis*, from the other *Burkholderia* species. The effective mass that can discriminate between *B. mallei*/*B. pseudomallei* group and *B. thailandensis* is m/z 6,551. Due to the close relationship between the species of *B. mallei* and *B. pseudomallei*, they have suggested that these two strains differ significantly, based on the mass peak intensity at m/z 5,794 and 7,553 by using ClinProTools software. As expected, the MALDI-TOF average mass spectra of all tested strains, in our study, contained the prominent biomarkers (m/z 4,410, 5,794, 6,551, 7,553, and 9,713) as shown in vertical dashed lines in Figure 1. It indicates that these mass ions are conserved as species-specific biomarkers among *B. pseudomallei* strains based on the whole-cell MALDI-TOF method.

**Table 1:** Identification scores of all *B. pseudomallei* isolates.

Isolate	Identification score
E10	1.90
E14	2.17
E239	2.26
E367	2.18
E390	2.29
K96243	2.48
PP844	2.28
NF10/38	1.94
NF47/38	1.90
NF105/37	2.22
NF154/37	1.99

### Cluster analysis of *B. pseudomallei* isolates

**Figure 2:**
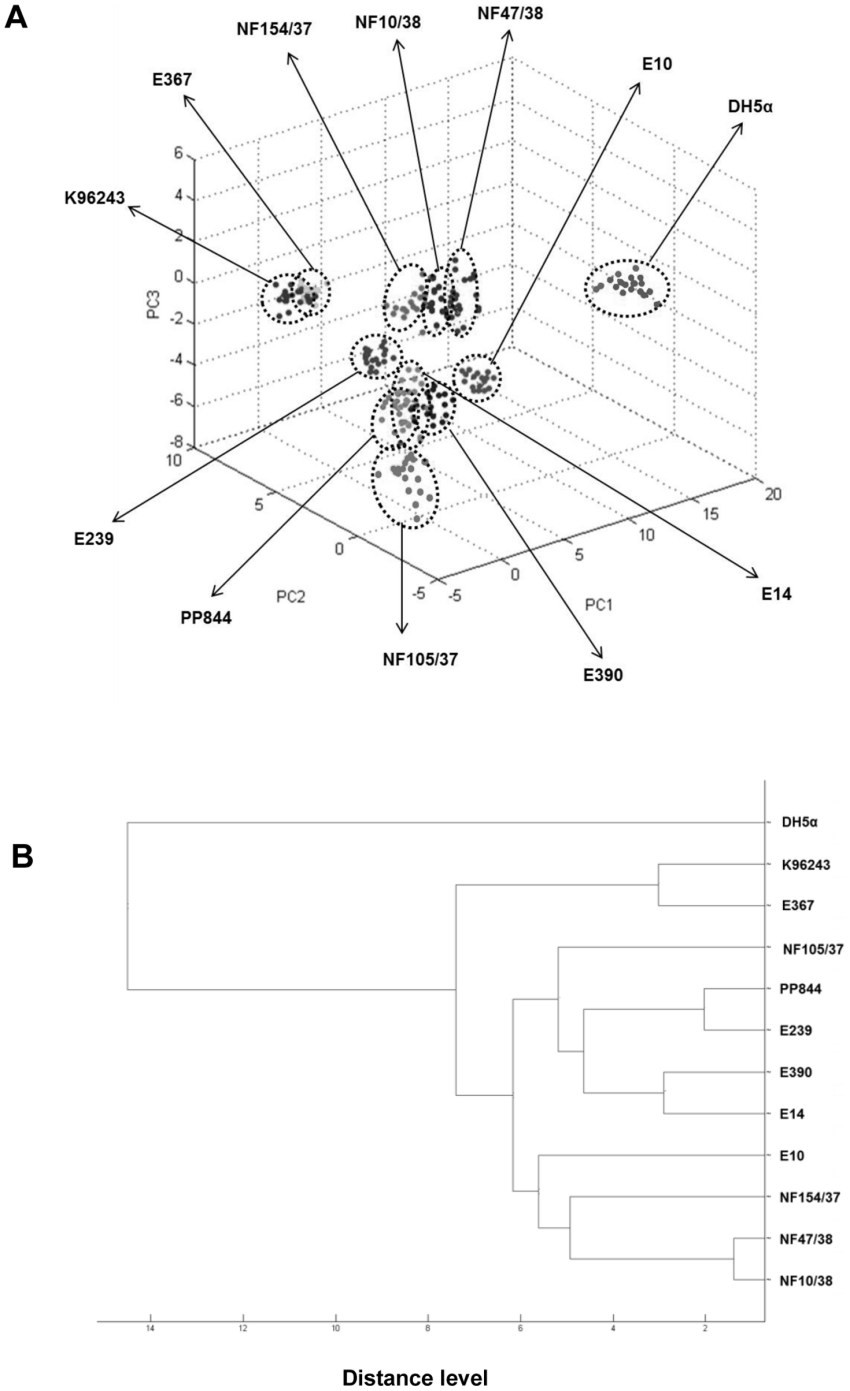
Principal component analysis (PCA) and cluster analysis of *B. pseudomallei* isolates. A) PCA analysis analyzed from total mass spectra showed intermixture and widely spreaded of all isolates from two source groups. **B**) Dendrogram constructed from the scores of PCA demonstrated that three of six environmental isolates; E239, E390, and E14 were grouped on their own cluster. Similarly, three clinical isolates, consisting of NF154/37, NF47/38, and NF10/38, were clustered together. *E. coli* DH5α was used as an outgroup taxon. Numbers shown in x-axis revealed distance level of tested isolates.

In this study, a total of eleven isolates of *B. pseudomallei* came from two major sources, environmental and clinical sources. To determine whether the ability of whole-cell MALDI-TOF MS method could group isolates according to their respective sources, raw mass spectra of all *B. pseudomallei* isolates, obtained from FlexAnalysis, were then analyzed using ClinProTools software to generate a principal component analysis (PCA) and a dendrogram. PCA demonstrated that environmental and clinical isolates intermixed together and did not form distinct groups according to their own origins (Figure 2A). Dendrogram calculated from the PCA scores, on the basis of a scored-based algorithm, illustrated that three of the six environmental isolates; E239, E390, and E14, were grouped on their own cluster, the same as the three clinical isolates; NF154/37, NF47/38, and NF10/38. Two of the clinical isolates; NF105/37 and PP844 were grouped with the environmental clade. The E10, from the environmental source, was grouped with the clinical cluster. In addition, K96243 and E367 isolates were dispersed and formed their own cluster (Figure 2B). Even though all of *B. pseudomallei* isolates were not clustered by their sources, most of them (6 out of 11 isolates) could be differentiated according to their respective source groups using the whole-cell MALDI-TOF MS method.

### Biomarkers for discrimination of environmental and clinical *B. pseudomallei* isolates

**Figure 3:**
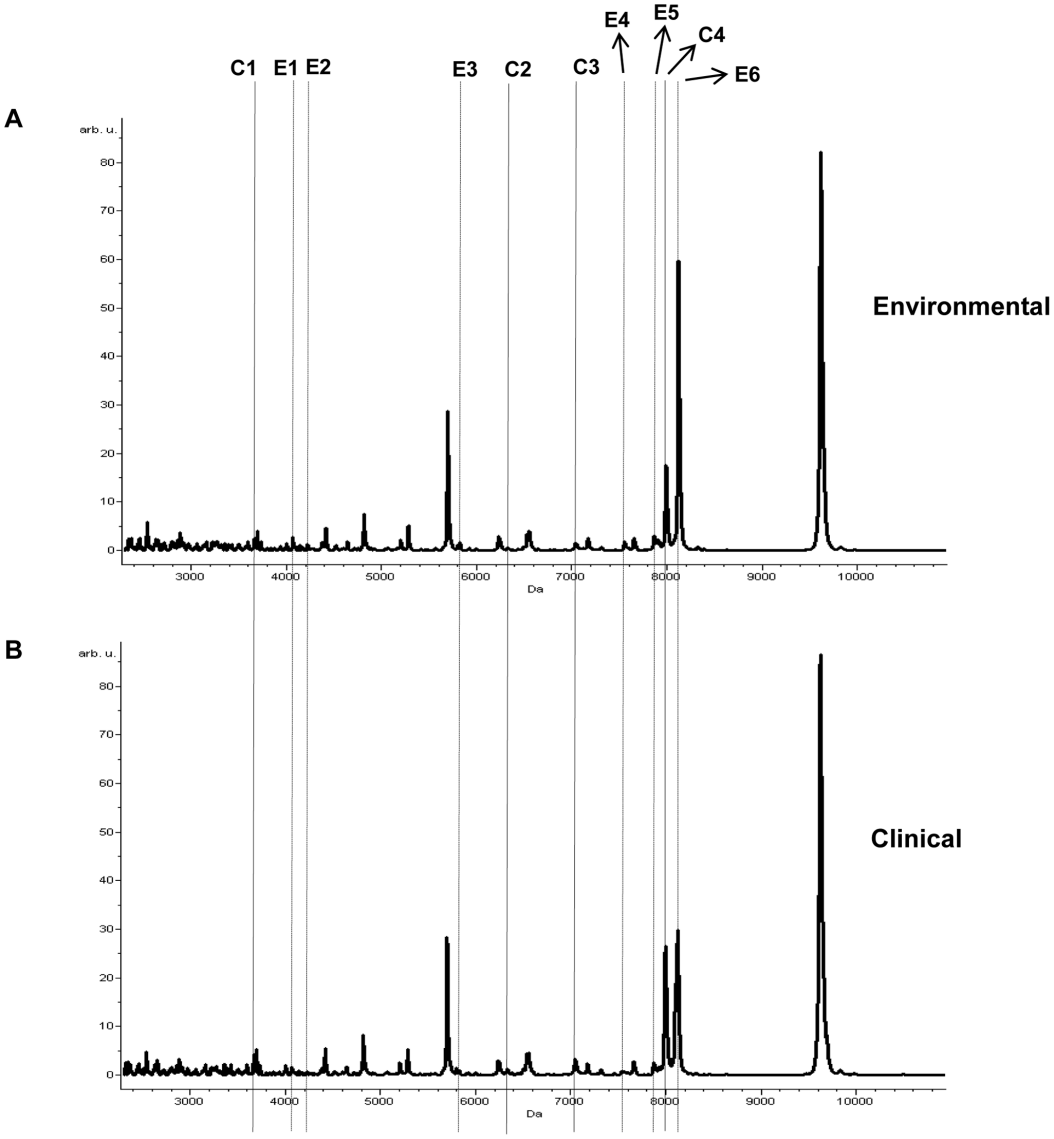
Potential source-identifying biomarker ions obtained from ClinProTools analysis. An average environmental spectrum of E10-E390 strains (**A**) had a very similar pattern as in a clinical set of K96243-NF154/37 (**B**) but peak intensity was different. The vertical dashed lines, E1-E6, indicated as environmental-identifying biomarker ions (EIBIs), consisting of m/z 4,056, 4,214, 5,814, 7,545, 7,895, and 8,112 whereas the vertical solid lines marked as C1-C4 displayed clinical-identifying biomarker ions (CIBIs), containing m/z 3,658, 6,322, 7,035, and 7,984.

An average mass spectrum of environmental set was constructed from a set of 5 environmental average spectra (E10-E390) (Figure 3A). Similarly, an average mass spectrum of clinical set was generated from a combination of 6 clinical average spectra (K96243-NF154/37) as displayed in Figure 3B. The two average mass spectra of these two groups demonstrated a high similarity in peak patterns but differed in peak intensities. To investigate the source-specific biomarkers for discrimination of *B. pseudomallei* corresponding to their origins (environmental and clinical sources), we analyzed these two source-representative mass spectra by using the Quick Classifier (QC) algorithm in the ClinProTools software which subsequently provided the candidate peak lists between the two sample groups based on statistical calculations, Wilcoxon/Kruskal-Wallis statistics. The potential source-specific biomarker peaks were evaluated in the mass range of m/z 2,000–20,000. ClinProTools analysis totally revealed 23 biomarkers that demonstrated significant differences in peak intensity between environmental and clinical groups (data not shown). We stringently examined further on differential peak signals between the two sets and calculated as fold differences of the individual biomarker. Peak intensity values and fold differences were summarized in Table 2. Fold difference of each biomarker was individually calculated using peak intensity of environmental divided by that of clinical group. Biomarkers that exhibited > 1.5 fold and < 0.67 fold were chosen and classified as environmental-specific and clinical-specific mass ions, respectively. A total of 10 effective source-specific biomarkers was therefore selected. Six out of the ten biomarkers were defined as environmental-identifying biomarker ions (EIBIs), consisting of m/z 4,056, 4,214, 5,814, 7,545, 7,895, and 8,112 (doubly charge of 4,056), which were shown as E1-E6, respectively (see the vertical dashed lines in Figure 3). While the remaining 4 biomarkers were assigned as clinical-identifying biomarker ions (CIBIs), which showed significantly higher intensities than that of the environmental set, containing m/z 3,658, 6,322, 7,035, and 7,984, which were marked as C1-C4, respectively (see the vertical solid lines in Figure 3). Hence, these mass ions could be used as the potential source-specific biomarkers to discriminate *B. pseudomallei* in relation to their source groups.

**Table 2:** Peak intensity values and fold differences of all source-identifying biomarker ions of *B. pseudomallei*.

Biomarker ion	m/z value	Peak intensity (arb.u.)		Fold difference*
		environmental	clinical	
E1	4,056	2.7	1.7	1.59
E2	4,214	1.2	0.7	1.71
E3	5,814	1.7	1.0	1.70
E4	7,545	1.8	0.8	2.25
E5	7,895	2.25	0.98	2.30
E6	8,112	59	29	2.03
C1	3,658	2.375	4.125	0.58
C2	6,322	0.6	1.325	0.45
C3	7,035	1.5	3.4	0.44
C4	7,984	16.5	26.3	0.63

All ten source-identifying biomarker ions were selected on the basis of Wilcoxon/Krustal-Wallis statistics which were significantly different at p < 0.01.

*Fold difference of each biomarker ion was calculated using peak intensity of environmental divided by that of clinical group.

## Discussion

Whole-cell MALDI-TOF MS has been demonstrated as a useful tool for rapid identification and classification of a variety species of microorganisms. It could be observed that most of the resultant mass peaks, as shown in *B. pseudomallei* mass fingerprints (Figure 1), were in the range of m/z 2,000–11,000 Da, which are typically reported to be adequate for species discrimination and are similar to the results of *Salmonella* and *Vibrio sp*. [32], [37]. Each average mass spectrum, belonging to each strain, exhibited very similar peak patterns but distinctive peak intensities (Figure 1). Due to our MALDI BioTyper database not containing the main spectral projections (MSPs) of *B. pseudomallei*, we had to construct an in-house *B. pseudomallei* strain K96243 as a reference spectrum for performing pattern matching and further identifying the tested samples at both genus and species levels. According to criteria for species identification, the score must be ≥ 1.90. As per the results in Table 1, the scores of a total of eleven tested isolates, obtained from BioTyper analysis, varied from 1.90–2.48, indicating that all the isolates in this study were confirmed as *B. pseudomallei*. The range of identification scores extensively varied suggesting a variation at the subspecies level of *B. pseudomallei*, in agreement with Karger's study, as they reported a large score ranging between 2.25–2.89 [41]. The results revealed lower identification scores than which of Karger's, owing to in this study we constructed only K96243 as a reference spectrum and all mass spectra (from a total of 11 *B. pseudomallei* strains) were queried to perform pattern matching against the K96243 reference spectrum. It was notably observed that K96243 had the highest score value because of the existing of K96243 reference spectrum in our in-house MSPs database. However, the obtained identification scores were still sufficient for confirmation procedure of all *B.pseudomallei* isolates based on MALDI-TOF. Not only availability of MSPs databases but also the influences of culture media and incubation times might have caused the lower identification scores. Our study used different medium and incubation time for bacterial cultivation, such as using Ashdown's selective agar for 7 days according to Chantratita et al [43] because it is a selective medium that is commonly used for isolating and culturing *B. pseudomallei*, which differed from Karger's study, which used a nutrient blood agar and incubated for 48 hours. The standard protocols for whole-cell MALDI-TOF must be maintained for reducing the data variation between laboratories. Nevertheless, the mass ions, defined as taxon-specific biomarkers for *B. pseudomallei* identification, were detected in our findings; including, m/z at 4,410, 5,794, 6,551, 7,553, and 9,713 [41]. In detail, the *Burkholderia* species must contain m/z 4,410 since it regards as a faithful biomarker among *Burkholderia* spp. The mass ion at m/z 9,713 is specific for the *Pseudomonas* group, including *B. mallei*, *B. pseudomallei*, and *B. thailandensis*. The mass peak at m/z 6,551 is an effective mass ion that is used for dividing *B. thailandensis* from the *B. mallei*/*B. pseudoma*llei group. As study from Lau et al, these taxon-specific biomarkers can also be found in MALDI-TOF MS mass spectrum of *B. pseudomallei* [44]. Hence, these five biomarkers are common and conserved as species-specific biomarkers among *B. pseudomallei* strains. At m/z 5,794 and 7,553, Karger et al have also suggested the consideration of peak intensity at these two m/z peaks for differentiating *B. mallei* and *B. pseudomallei*, because these two species are closely related. We observed likely mass ions at m/z 5,794 resulting in the higher peak intensity than the m/z 7,553 in our tested *B. pseudomallei* samples. Therefore, it could be inferred that the whole-cell, or intact cell, method should be able to be used to analyze the samples under different experimental conditions and generate the stable mass ions for bacterial identification analysis, as previously described in several studies [26], [45].

Our principal component analysis (PCA) demonstrated that all tested *B. pseudomallei* isolates were intermixed, thus they were not grouped in accordance with their origins (Figure 2A). The PCA scores were then used to generate a dendrogram in order to examine the ability of MALDI-TOF MS in grouping analysis of samples from different sources. From a total of 11 strains, overall six strains were grouped correctly to their sources (Figure 2B). In this study, whole-cell MALDI-TOF MS seems likely to possess low ability of categorization with *B. pseudomallei* samples. However, the MALDI-TOF MS application for cluster analysis by source is widely used with other bacterial species, such as *E. coli* [30], and *Entercoccus* spp. [33]. In those studies, the rates of overall correct classification of *E. coli* and *Enterococcus* spp. by MALDI-TOF MS are 73% and 67%, respectively. Those authors have further suggested that grouping analysis on the basis of the MALDI-TOF approach does not classify all of isolates correctly with respect to their own sources (for example, it does not succeed in the grouping of *E. coli and Enterococcus* isolated from humans), however, MALDI-TOF still represents an effective ability in grouping analysis when compared to other molecular typing methods, such as rep-PCR [30], [33]. Therefore, examination in terms of source categorization by MALDI-TOF could be altered, depending on a variety of tested bacterial species. In our results, the imprecise cluster analysis on the basis of the mass fingerprint-based approach between these two source groups of *B. pseudomallei* strains might indicate the relatedness of clonality between environmental and clinical isolates as suggested by Haase et al and Currie et al [11], [46]. In addition, there is a chance that the occurrence of invasive *B. pseudomallei* strains from clinical specimens, such as fecal, urine, sputum, and pus of infected humans or animals can contaminate the environment, followed by infection in humans and animals, that are exposed to contaminated soil and water [47]. These events could lead to the misclassification of *B. pseudomallei* from differential source groups. The small number of samples in this study might also provide less information for strain categorization using MALDI-TOF analysis. A collection from *B. pseudomallei* of each source should be added and other experimental factors that affect the quality of mass spectra have to be considered and examined in future experiments in order to obtain sufficient peak ions for use in cluster analysis.

Several publications have previously illustrated the use of various molecular typing methods for both the identification and differentiation of *B. pseudomallei* strains according to their different sources [14]–[20], [48]. Bartpho et al have recently applied the microarray-based comparative genome hybridization (CGH) method to discover different genomic islands (GIs) in environmental and clinical samples and have found that clinical *B. pseudomallei* isolates contain GI8.1, 8.2, and 15, which cannot be detected in environmental isolates [21]. But the limitations of these techniques are that they are time-consuming, labour- and cost-intensive and require several steps to accomplish in the process of the identification and typing of microorganisms. To our knowledge, this study is the first to describe the use of whole-cell MALDI-TOF MS to identify the source-identifying biomarker ions of environmental and clinical *B. pseudomallei* isolates at the proteomic profiling level. Average environmental and clinical mass spectra which were source representatives demonstrating a high level of peak pattern similarity but differed in peak intensity were further analyzed by ClinProTools software to discover the potential source-specific biomarkers in the mass range of 2,000–20,000 Da. We exhibited a total of ten mass ions corresponding to source-identifying biomarkers that showed significant differences of peak intensity between environmental and clinical *B. pseudomallei* groups (Table 2). Six environmental-identifying biomarker ions (EIBIs) including, m/z 4,056, 4,214, 5,814, 7,545, 7,895, and 8,112 (doubly charge of 4,056), showed an obvious higher peak intensity than those in the clinical set. While four clinical-identifying biomarker ions (CIBIs), consisted of m/z 3,658, 6,322, 7,035, and 7,984, explicitly contained higher mass intensity over that of the environmental set (Figure 3). For obtaining sufficient peak profiles, suitable protocols for whole-cell MALDI-TOF analysis and other important factors, including bacterial culture conditions, matrix types, sample preparation methods, and variabilities in crystal formation, which influence the mass fingerprints in respect to peak quality and quantity, have to be investigated for an improvement on capability to distinguish closely-related species at the strain level. Typically, several researchers have shown that the protein biomarkers for identification at genus- and species-level correspond to abundant proteins inside the cells, such as ribosomal proteins and DNA or RNA binding proteins [23], [28], [35]. Dieckmann et al have additionally found that mass peak ions, which are subspecies-specific biomarkers in *Salmonella* spp., also contain putative uncharacterized proteins, thus the means of strain identification of bacteria requires the consideration of the extent of all those proteins in addition to ribosomal proteins [32]. Our study has yet to assign all EIBIs and CIBIs of *B. pseudomallei* to that of known expressed proteins. Further mass peak identification based on a bioinformatics-enabled approach could provide more information of differentially expressed proteins among environmental and clinical isolates under certain conditions.

## Materials and Methods

### Bacterial isolates and culture conditions

All bacterial isolates were cultured under the BSL3 conditions, which are approved by the committee from Faculty of Science, Mahidol University. The bacteria were provided by the authorities via personal permission. Environmental *B. pseudomallei* samples were isolated from soil in various areas of northeast Thailand and clinical samples were obtained from melioidosis patients which were stored in 80% glycerol stock at −80°C. Isolation sources of all *B. pseudomallei* samples were shown in Table 3. Each bacterial sample from glycerol stock was cultured in Luria-Bertani (LB) broth medium for 16 hours at 37°C with shaking at 180 rpm. Subsequently, the bacteria were subcultured into a new LB broth medium with 0.1% inoculum and incubated for 3 hours with shaking. To obtain colonies for MALDI-TOF MS analysis; after incubation the bacterial culture was serially diluted with LB medium, spread plated on Ashdown's selective agar and incubated at 37°C for 7 days as previously described in [43].

**Table 3:** All *Burkholderia pseudomallei* strains used in this study.

Source	Isolate	Isolation source	Reference
Environmental	E10^a^	Ubon Ratchathani	[51]
	E14^a^	Ubon Ratchathani	[51]
	E239^a^	Yasothon	this study
	E367^a^	Si Sa Ket	this study
	E390^a^	Ubon Ratchathani	this study
Clinical	K96243^b^	Pus	[49]
	PP844^c^	Blood	[53]
	NF10/38^d^	Blood	[52]
	NF47/38^d^	Blood	[52]
	NF105/37^d^	Pus	[52]
	NF154/37^d^	Pus	[52]

a E10-E390 were received from Dr. Vanaporn Wuthiekanun, Wellcome Trust Unit, Faculty of Tropical Medicine, Mahidol University, Bangkok, Thailand.

b K96423 was received from Assoc. Dr. Surasakdi Wongratanacheewin, Melioidosis Research Center, Faculty of Medicine, Khon Kaen University, Khon Kaen, Thailand.

c PP844 was received from Prof. Dr. Stitaya Sirisinha, Department of Microbiology, Faculty of Science, Mahidol University, Bangkok, Thailand.

d NF10/38-NF154/37 were received from National Institute of Health of Thailand, Ministry of Public Health, Nonthaburi, Thailand.

### Sample preparation for MALDI-TOF MS analysis

The bacterial colonies on Ashdown's agar were picked into 900 µl of water and suspended with 300 µl of ethanol. Bacterial cells were harvested by centrifugation. The cell pellets were then resuspended and vigorously mixed with MALDI matrix solution consisting of 10 mg sinapinic acid in 1 ml of 50% acetonitrile containing 2.5% trifluoroacetic acid. A 2-μL of the mixture was directly spotted onto a MALDI target plate (MTP 384 ground steel plate, Bruker Daltonik, GmbH, Bremen, Germany) and allowed to air dry. Twenty replicates (n = 20) of each bacterial lysate were spotted onto the target plate in the same mass spectrometer run in order to examine data reproducibility.

### MALDI-TOF MS instrument and data analysis

A Ultraflex III TOF/TOF mass spectrometer (Bruker Daltonik GmbH, Bremen, Germany) was employed for sample analysis. The instrument was externally calibrated using a ProteoMass peptide & protein MALDI-MS calibration kit (Sigma-Aldrich, St. Louise, MO) which includes human ACTH fragment 18–39 (m/z  =  2,465), bovine insulin oxidized B chain (m/z  =  3,465), bovine insulin (m/z  =  5,731), equine cytochrome c (m/z  =  12,362), and equine apomyoglobin (m/z  =  16,952). MALDI-TOF MS operated in linear positive mode within the mass range of 2,000–20,000 m/z. Five hundred shots were accumulated, with a 50 Hz laser, for each sample. Parameters in flexControl were carried out according to the manufacturer's instructions (Bruker Daltonik GmbH, Bremen, Germany) using acceleration voltage of 25.00 kV (ion source 1) and 23.45 kV (ion source 2) and lens voltage of 6.0 kV. On account of our BioTyper database not containing *B. pseudomallei* reference spectra, we therefore constructed mass spectra of strain K96243 as an in-house reference spectrum, because this strain is well-known and is the first of the established genome sequences [49]. The reference spectrum of K96243 was generated from twenty single spectra. Total mass spectra of each bacterial sample acquired from FlexAnalysis version 3.0 (Build 92) were used to perform pattern matching against the reference spectrum of the K96243 strain using the MALDI BioTyper 2.0 software package. The calculation of matching peaks between the tested spectra and the reference spectra in the database provides the identification score for any given sample. Average spectrum for each *B. pseudomallei* strain was constructed from a set of twenty replicate spectra. For identifying the biomarkers to discriminate between environmental and clinical stains, the two average mass spectra of these two groups were further analyzed using ClinProTools version 2.2 (Build 78) [50]. Parameter settings for spectra preparation in the ClinProTools software were: top hat baseline subtraction, a resolution of 800 ppm, and a mass range of 2,000–20,000 m/z. Peak picking was based on total average spectrum, using a signal to noise ratio threshold of 5. The sort mode for peak selection was based on Wilcoxon/Kruskal-Wallis statistics at p-value < 0.01. Principal component analysis (PCA) and dendrogram for cluster analysis were automatically conducted with the integrated tool in the ClinProTools, MATLAB algorithm.

## Conclusions

Whole-cell MALDI-TOF MS is a worthy and rapid tool which can be employed to analyze bacterial isolates under different experimental conditions and generate the stable mass ions for reliable bacterial identification analysis. PCA and cluster analysis demonstrated that environmental and clinical isolates of *B. pseudomallei* intermixed together and could not completely group all isolates in accordance to their sources. Future experiments should comprise a large number of *B. pseudomallei* from each source group to obtain more mass spectra information for the improvement of source categorization analysis. Notably, our study has shed light on the efficacy of whole-cell MALDI-TOF analysis for identifying the source-specific biomarkers for facilitating the discrimination of environmental and clinical *B. pseudomallei* isolates. The rapid typing of *B. pseudomallei* from various sources, using the MALDI-TOF approach, could enable the tracking of *B. pseudomallei* during outbreaks and provide benefits with regards to medical prevention and the treatment of melioidosis. To the future aspects, a bioinformatics-based approach should be applied for investigating protein assignment and providing powerful identification and differentiation procedures of microorganisms at the strain level.
